# Survival of esophageal squamous cell carcinoma achieving pathological complete response after neoadjuvant immunochemotherapy

**DOI:** 10.1007/s00432-026-06587-6

**Published:** 2026-08-12

**Authors:** Zongpu Zhang, Bin Li, Qingyuan Huang, Tao Yu, Longlong Shao, Junjie Lv, Longfei Ma, Longsheng Miao, Yawei Zhang, Yang Zhang, Hong Hu, Jiaqing Xiang, Haiquan Chen

**Affiliations:** 1 Department of Thoracic Surgery and State Key Laboratory of Genetics and Development of Complex Phenotypes, Fudan University Shanghai Cancer Center, Shanghai, 200032 China; 2 Institute of Thoracic Oncology, Fudan University, Shanghai, 200032 China; 3 Department of Oncology, Shanghai Medical College, Fudan University, Shanghai, 200032 China

**Keywords:** Esophageal cancer, Squamous cell carcinoma, Immunochemotherapy, Pathological complete response, Disease-free survival

## Abstract

**Background:**

Immunochemotherapy is increasingly adopted as neoadjuvant regimen for locally advanced esophageal squamous cell carcinoma (ESCC). This study aimed to evaluate the survival outcomes for those who achieved pathological complete response (pCR) following immunochemotherapy.

**Methods:**

From January 2020 and June 2025, 154 patients who achieved pCR after neoadjuvant immunochemotherapy were included for this analysis. We documented baseline patient characteristics, assessed survival outcomes, and designated disease-free survival (DFS) as the primary endpoint.

**Results:**

Of the 154 patients, there were 47 (30.5%) patients received chemotherapy combined with pembrolizumab, 37 (24.0%) with tislelizumab, 31 (20.1%) with sintilimab, 26 (16.9%) combined with camrelizumab, and 13 (8.4%) with other immune checkpoint inhibitors. Majority of the patients (74.1%, 114/154) had two treatment cycles. There were 81 patients (52.6%) underwent McKeown procedure and 73 (47.4%) underwent Ivor Lewis procedure, with the median of 30 lymph nodes resected. After a median follow-up of 25 months, the 1-year and 3-year DFS were 97% and 78%, respectively, the 1-year and 3-year overall survival were 97% and 88%, respectively. Advanced clinical tumor stage (hazard ratio [HR] = 4.831, 95% confidence interval [CI] 1.748–13.347, P = 0.002), more than two treatment cycles, (HR = 3.449, 95% CI 1.168–10.179, P = 0.025), and a treatment-to-surgery less than 6 weeks (HR = 5.344, 95% CI 1.198–23.840, P = 0.028) were associated with reduced DFS.

**Conclusion:**

ESCC patients achieving pCR after immunochemotherapy had favorable survival outcomes, yet disease recurrence remains a notable concern. Advanced tumor stage, increased number of treatment cycles and shortened interval between neoadjuvant treatment and surgery were associated with compromised survival in this patient population. These exploratory findings may help guide future prospective studies.

## Introduction

Neoadjuvant therapy followed by curative esophagectomy has been recommended for patients with locally advanced esophageal squamous cell carcinoma (Ken et al. 2024; Qin et al. 2024; Eyck et al. 2021). However, there is still no consensus regarding the optimal treatment regimen. As immunotherapy combined with chemotherapy significantly improved patient survival as the first-line treatment for esophageal cancer (Lu et al. 2022; Sun et al. 2021), neoadjuvant immunochemotherapy, consequently, has been increasingly used for resectable tumor.

The safety and efficacy of neoadjuvant immunotherapy have been proved in published studies (Qin et al. 2024). The rates of pathologically complete response (pCR) were ranging from 16.7 to 50% (Xu et al. 2022). However, disease recurrence is still common among those who achieved pCR. Hence, the aim of this study was to comprehensively document the prognosis of the patients who achieved pCR after neoadjuvant immunochemotherapy, and to analyze the associated risk factors to guide future clinical work.

## Methods

### Patients

**Fig. 1:**
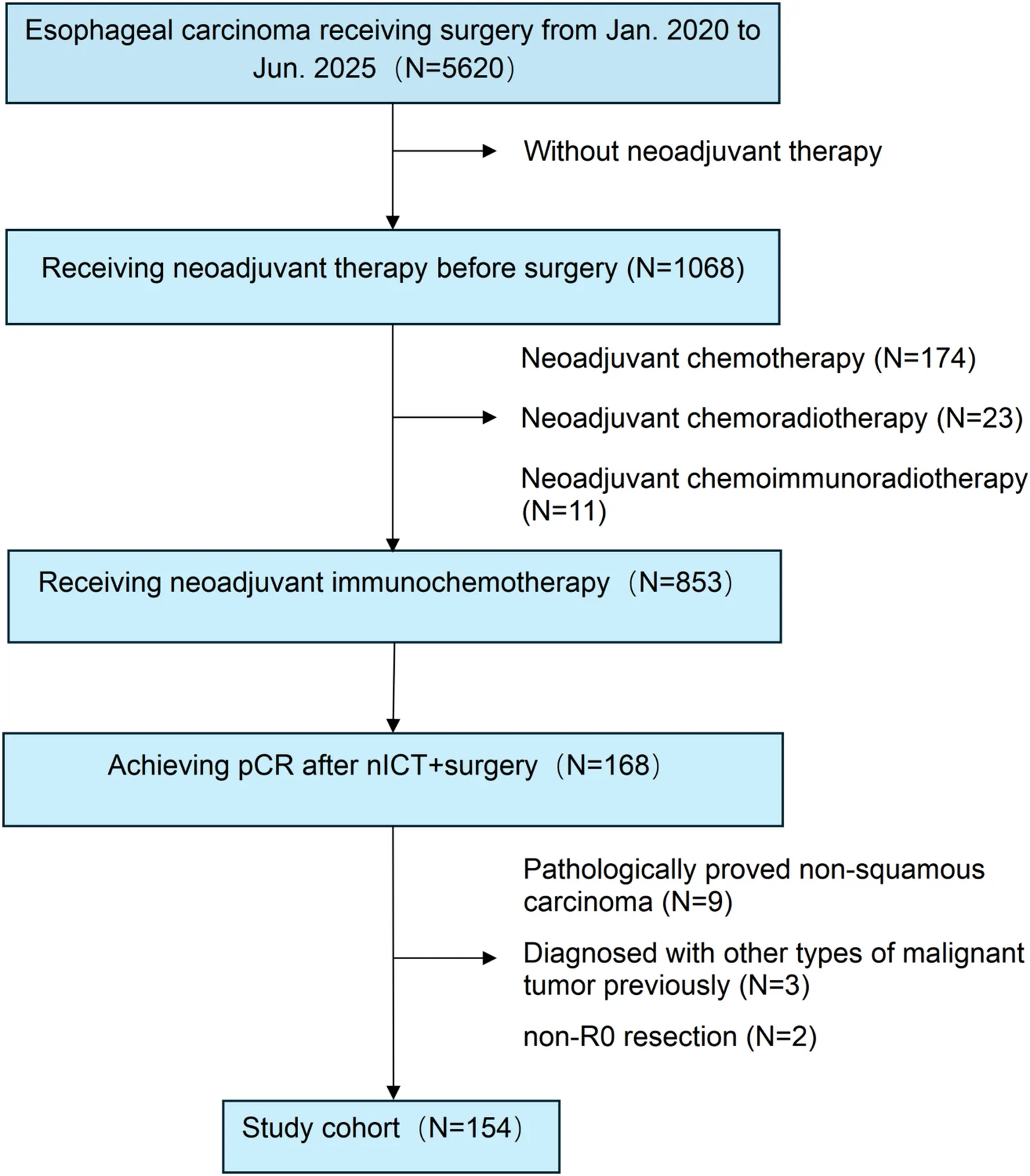
Flowchart of patient enrollment and study selection

Between January 1st, 2020 and June 30th, 2025, a total of 5620 patients underwent esophagectomy due to cancer at Fudan University Shanghai Cancer Center, of which 1068 received preoperative therapy due to advanced tumor stage and 853 had immunochemotherapy, among which 168 achieved pCR, with a pCR rate of 19.7% (168/853) during the study time (Fig. 1). Additional review of all pCR patients further excluded 9 patients with non-squamous carcinoma, 3 patients previously diagnosis with other malignancy and 2 patients with non-R0 resection. Ultimately, 154 patients were included in this retrospective analysis. Briefly, all the records of patients who had preoperative therapy were reviewed, and the inclusion criteria for this analysis were as follows: (1) esophageal squamous cell carcinoma by pretreatment endoscopic biopsy; (2) neoadjuvant chemotherapy combined with immunotherapy; (3) no radiotherapy prior to surgery; (4) no history of other malignancy; (5) pathologic complete response by pathological confirmation and (6) R0 resection (complete resection of the primary tumor and regional lymph nodes with no microscopic or macroscopic residual disease).

The institutional review board of Fudan University Shanghai Cancer Center approved the use of database for this study (No. 1612167-18). Informed consent was taken from all the patients. The study was conducted in accordance with the Declaration of Helsinki.

### Treatment

Preoperative workup assessing patient operability included a complete history, physical examination, endoscopy of the entire upper gastrointestinal tract, histologic confirmation of the carcinoma, ultrasonography of the neck, computed tomography (CT) of the chest and abdomen. Pulmonary function testing and electrocardiography were also performed to assess fitness. Positron emission tomography/computed tomography has been routinely performed after 2023 in Shanghai Cancer Center.

Immunochemotherapy was always applied to those with tumor of clinical stage T1-2N1-3M0 and T3-4aN0-3M0. As there is still no consensus on the optimal neoadjuvant regimen, different types of immune checkpoint inhibitors were used based on surgeons’ experience and preference in China. Pembrolizumab, tislelizumab, camrelizumab, and sintilimab were commonly used in this cohort. Of the 154 patients, there were 100 patients (64.9%) had immunotherapy in our hospital, and 54 (35.1%) in their local hospitals, of whom the preoperative examinations were unavailable.

Surgery was scheduled 4 to 6 weeks after the treatment. The McKeown procedure and Ivor-Lewis procedure, via open or minimally invasive approach, were performed depending on the surgeon’s preference. Extended radial two-field lymphadenectomy is required with curative intent. When lymph node metastasis in the neck was indicated on CT or ultrasonography, cervical lymphadenectomy was performed. Patients were staged according to the tumor-node-metastasis classification of the eighth edition of the American Joint Committee for Cancer Staging Manual (Rice et al. 2017).

Morbidity and mortality were examined within 90 days of surgery. Postoperative complications were recorded, including anastomotic leakage, pulmonary infection, pulmonary embolism, chylothorax, and other complications (intestinal obstruction, wound infection, and vocal cord paralysis).

For patients underwent neoadjuvant immunochemotherapy, postoperative treatment strategy remains undefined. Routine follow-up was recommended in recent clinical practice. During the early phase of this study, adjuvant immunochemotherapy or single-agent immunotherapy was determined based on by the surgeons’ clinical judgement and patients’ postoperative recovery status.

### Histopathological assessment, follow-up and outcomes

Esophageal resection specimens were histopathologically evaluated by experienced pathologists specializing in esophageal cancer. During the original pathological examination, the tumor type, differentiation grade, depth of invasion, number of involved lymph nodes, and presence of lymphovascular invasion were recorded. Sample margins, including the circumferential resection margin, were also recorded. In this study, pCR was defined as no residual tumor in both the primary tumor and lymph nodes.

After surgery, patients underwent assessments every three months for the first two years, followed by every six months for the next three years, and annually thereafter. Survival status, and disease progression were documented at each follow-up, which included a combination of outpatient visit records, hospitalization logs, and telephone interviews. Routine evaluations comprised a comprehensive physical examination, cervical ultrasound, and thoracic combined with abdominal contrast-enhanced CT scans. When clinically indicated, PET/CT and endoscopic ultrasound-guided biopsies were also performed. In this study, the primary outcomes were disease-free survival (DFS).

### Statistical analysis

Data were presented as numbers and percentages, means and standard errors, or medians and interquartile ranges. DFS was defined as the time from surgery to tumor recurrence, death, or the last follow-up. Overall survival (OS) was measured from surgery to death from any cause or the last follow-up. Prognostic analyses for OS were not performed due to the limited number of events. Survival outcomes were established and compared using Kaplan–Meier curves and the log-rank test. Cox proportional hazards regression was employed to assess prognostic factors for DFS. Univariable Cox regression analyses were performed for all candidate variables, including age, sex, BMI, tumor location, clinical T stage, clinical N stage, cycle of neoadjuvant therapy, treatment-to-surgery interval, surgery procedure, surgical approach, number of lymph nodes resected, and adjuvant therapy. Variables with a P-value < 0.15 in the univariable analysis were selected for inclusion in the multivariable Cox regression model. This threshold was chosen to avoid excluding potentially important variables due to weak effects in small-sample settings. Results are presented as hazard ratios (HRs) with 95% confidence intervals (CIs). A two-sided P-value < 0.05 was considered statistically significant. All data were analyzed with SPSS version 20.

## Results

### Patients’ characteristics

Of the 154 patients, the mean age was 62 ± 7 years. There were 125 male (81.2%) patients and 69 (44.8%) had the tumors located in the middle thoracic esophagus. All patients received platinum-based chemotherapy, and there were 47 (30.5%) received chemotherapy combined with pembrolizumab, 37 (24.0%) with tislelizumab, 31 (20.1%) with sintilimab, 26 (16.9%) combined with camrelizumab, and 13 (8.4%) with others. Regarding treatment cycle, there were 1 patient (0.6%) had one treatment cycle, 114 (74.1%) had two cycles, 23 (14.9%) had three cycles, and 16 (10.4%) had four cycles. The median time from the completion of treatment to surgery was 34 days (interquartile range [IQR], 30–40). Of the 100 patients who had treatment in Shanghai Cancer Center, majority had clinical T3 (51, 51%) or N1 (54, 54%) disease before treatment (Table 1).

**Table 1:** Patient characteristics of 154 patients

Characteristics	Values
Age (year)	62 ± 7
*Gender*	
Male	125 (81.2)
Female	29 (18.8)
BMI	22.7 (20.8–24.7)
Hypertension	34 (22.1)
Diabetes	14 (9.1)
Smokers†	92 (59.7)
Drinkers‡	82(53.2)
*Tumor locations*	
Upper	23 (14.9)
Middle	69 (44.8)
Lower	62 (40.3)
*ASA fitness grade*	
1	8 (5.2)
2	142 (92.2)
3	4 (2.6)
*Immune checkpoint inhibitors*	
Pembrolizumab	47 (30.5)
Camrelizumab	26 (16.9)
Tislelizumab	37 (24.0)
Sintilimab	31 (20.1)
Other	13 (8.4)
*Cycle of neoadjuvant therapy*	
1	1 (0.6)
2	114 (74.1)
3	23 (14.9)
4	16 (10.4)
Treatment-to-surgery time	34 (30–40)
*Clinical T-stage*^*§*^	
2	21 (21)
3	51 (51)
4a	28 (28)
*Clinical N-stage*^*§*^	
0	11 (11)
1	54 (54)
2	27 (27)
3	8 (8)

Data are shown as mean ± standard error or n (%) or median (IQR). †, at least ten cigarettes per week for more than 6 months, including former and current smoker; ‡, any alcoholic beverage containing at least 20 g ethanol per week for more than 6 months, including former and current drinkers; § Clinical stage before immunochemotherapy (based on baseline imaging; n = 100 with complete imaging data). BMI, body mass index; ASA, American Society of Anaesthesiologist

Of the 154 patients, there were 81 patients (52.6%) underwent McKeown procedure and 73 (47.4%) underwent Ivor Lewis procedure. Majority of the surgeries were completed using open thoracic approach (57.1%). The median number of lymph nodes resected was 30 (IQR, 23–40). The most common complication after surgery in this cohort was pneumonia (12.3%), following by anastomotic leak (6.5%). No re-operation and hospital death in this cohort (Table 2).

**Table 2:** Surgical outcomes and adjuvant therapy of 154 patients

Surgical outcomes & adjuvant therapy	Values
*Surgery procedure*	
Mckeown	81 (52.6)
Ivor Lewis	73 (47.4)
*Surgical approach*	
Minimally invasive	66 (42.9)
Open	88 (57.1)
*Extent of lymphadenectomy*	
Three-field	14 (9.1)
Two-field	140 (90.9)
Operative time, mins	240 (200–292)
*Postoperative complications*	
Anastomotic leak	10 (6.5)
Pneumonia	19 (12.3)
Intestinal Obstruction	1 (0.7)
Recurrent Laryngeal Nerve Injury	3(1.9)
Chylothorax	3 (1.9)
Wound infection	1 (0.7)
Pulmonary Embolism	1 (0.7)
Re-operation	0
Hospital death	0
Number of lymph nodes resected	30 (23–40)
Hospital stay, days	13 (10–15)
Adjuvant therapy*	
Immunotherapy	42 (27.3)
Chemotherapy	5 (3.2)
Immunochemotherapy	17 (11.0)
No therapy	90 (58.4)

Data are shown as n (%) or median (IQR). *, adjuvant therapy excluding any treatment administered after recurrence

A total of 64 patients (41.6%) received postoperative adjuvant treatment: 42 (27.3%) were administered single-agent immunotherapy, 17 (11.0%) received immunochemotherapy, and 5 (3.2%) underwent adjuvant chemotherapy alone.

### DFS and OS

**Fig. 2:**
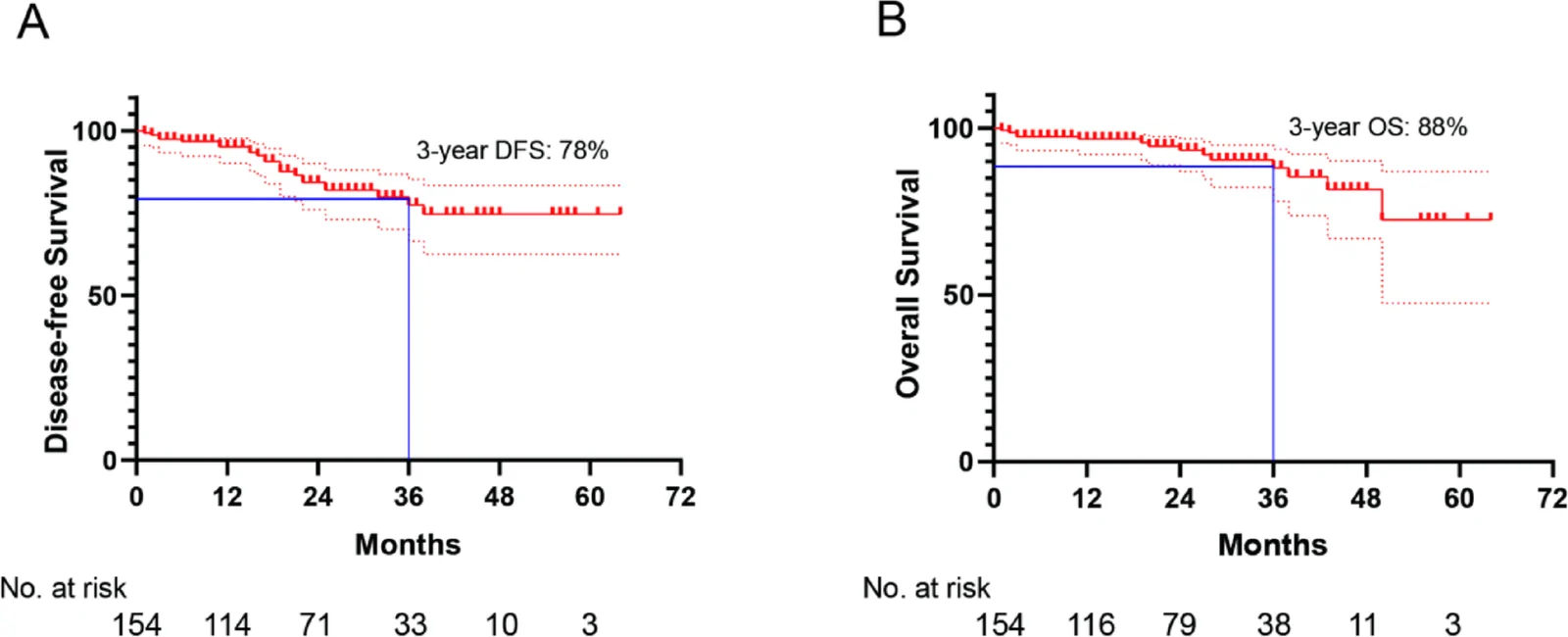
**Kaplan-Meier survival analyses of patients achieving pathological complete response (pCR) following neoadjuvant immunochemotherapy. A** Disease-free survival of patients with pCR after neoadjuvant immunochemotherapy. **B** Overall survival of patients with pCR after neoadjuvant immunochemotherapy

The median follow-up for the surviving patients was 25 months (IQR, 12–35 months). As of the last follow-up, recurrence was observed in 20 patients, and there were 14 deaths of the 154 patients, 11 of which were due to relapse of the disease. The cumulative 1-year DFS was 97% and 3-year DFS was 78% (Fig. 2A). The cumulative 1-year OS was 97% and 3-year OS was 88% (Fig. 2B).

Univariable Cox regression analyses showed that treatment-to-surgery interval (HR = 3.577, 95% CI 1.215–10.531, P = 0.021), clinical T stage (HR = 3.414, 95% CI 1.360–8.572, P = 0.009), and number of lymph nodes resected (HR = 1.028, 95% CI 1.002–1.055, P = 0.036) were associated with DFS, while cycle of neoadjuvant therapy (HR = 1.949, 95% CI 0.843–4.503, P = 0.119) met the P < 0.15 threshold for multivariable entry (Supplementary Table S1). The multivariable Cox proportional hazards model, restricted to the 100 patients with complete imaging data, revealed that advanced clinical T stage (HR = 4.831, 95% CI 1.748–13.347, P = 0.002), three or more treatment cycles (HR = 3.449, 95% CI 1.168–10.179, P = 0.025), and a treatment-to-surgery interval of less than 6 weeks (HR = 5.344, 95% CI 1.198–23.840, P = 0.028) were associated with reduced DFS, while the number of lymph nodes resected was not statistically significant (HR = 1.019, 95% CI 0.992–1.046, P = 0.174) (Table 3).

**Table 3:** Multivariate analysis of disease-free survival in all patients with complete image data (N = 100)

Characteristics	Multivariate analysis	
	Hazard ratio (95% CI)	P
Clinical T stage (T4a/T3/T2)	4.831 (1.748–13.347)	***0.002***
Cycle of neoadjuvant therapy(> 2/ < = 2)	3.449 (1.168–10.179)	***0.025***
Treatment-to-surgery weeks (< = 6/ > 6)	5.344 (1.198–23.840)	***0.028***
No. of lymph nodes resected (per 1 node increase)	1.019 (0.992–1.046)	0.174

Data were based on the 100 patients with complete imaging data. P < 0.05 is indicated by bold italics

**Fig. 3:**
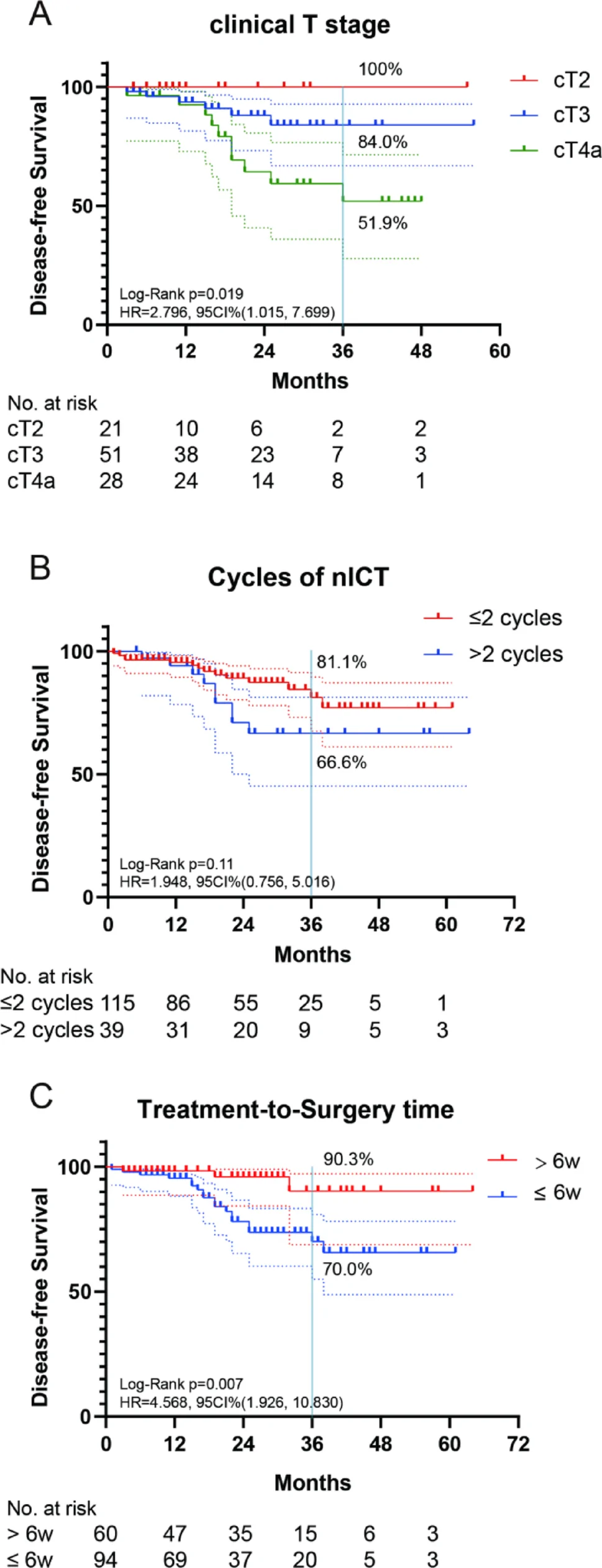
**Disease-free survival (DFS) of patients stratified by different clinicopathological and treatment-related factors. A** Disease-free survival of patients stratified by clinical tumor stage. **B** Disease-free survival of patients stratified by neoadjuvant treatment cycles. **C** Disease-free survival of patients stratified by interval between neoadjuvant treatment and surgery

Kaplan–Meier analyses stratified by the three factors identified in the multivariable model are shown in Fig. 3. For clinical T stage, the 3-year DFS rates were 100% for cT2, 84.0% for cT3, and 51.9% for cT4a (log-rank P = 0.019), based on the 100 patients with complete imaging data (Fig. 3A). For neoadjuvant treatment cycles, the 3-year DFS rates were 81.1% for ≤ 2 cycles and 66.6% for > 2 cycles (log-rank P = 0.11), based on the full cohort of 154 patients (Fig. 3B). For treatment-to-surgery interval, the 3-year DFS rates were 90.3% for > 6 weeks and 70.0% for ≤ 6 weeks (log-rank P = 0.007) (Fig. 3C).

## Discussion

It is widely accepted that patient survival is better among those who achieved pCR than those with tumor residual after neoadjuvant treatment. However, among patients who achieved pCR, postoperative outcomes varied considerably by neoadjuvant treatment modality (Okui et al. 2026). In this study, the 1 year-DFS was 97%, 3-year DFS was 78%, and majority of recurrence occurred within the first 3 years after surgery. Our findings identified pre-treatment clinical tumor stage, the number of neoadjuvant treatment cycles, and the interval between the last treatment and surgery as key factors associated with survival in this patient population.

Reported pCR rates vary among different studies (Xu et al. 2022). Different patient characteristics, and different treatment regimens used could lead to the different results. Notably, the extent of lymph nodes resected could also have an impact on the evaluation. The greater the number of lymph nodes examined, the higher the accuracy of disease staging. In this cohort, all patients had radical lymphadenectomy with the median number of lymph node resected of 30. Based on our previous study, three-field lymphadenectomy was applied for those with suspicious lymph node metastasis in the neck (Li et al. 2020, 2021). In this cohort, all patients underwent R0 resection with radical lymphadenectomy, and those with non-R0 resection were excluded to ensure accurate pCR evaluation. The pCR rate in this cohort was consistent with our previous report (Wu et al. 2021).

Disease recurrence is still not rare among those who achieved pCR. In a retrospective analysis using the National Cancer Database, majority of the patients had neoadjuvant chemoradiotherapy, showed that patients with advanced-stage esophageal cancer who achieved pCR remain at higher risk for recurrence (Harris et al. 2025). Our results showed similar results among patients who had neoadjuvant immunochemotherapy. For patients who had residual tumor after neoadjuvant chemoradiotherapy, Checkmate 577 study proved that adjuvant nivolumab improved patient survival (Kelly et al. 2021). Whereas among patients who had neoadjuvant immunochemotherapy, adjuvant immunotherapy provided no survival benefit in the overall cohort but improved survival among those with residual tumor (Chen et al. 2026). In this cohort, 64 of the 154 patients (41.6%) had adjuvant therapy, which was not associated with improved DFS. however, more studies are needed to confirm the value of adjuvant therapy among those with higher risks for recurrence.

In this research, treatment cycles, and the time interval between last treatment and surgery could also be associated with DFS. Fang et al. found a prolonged surgical interval exceeding 8 week following neoadjuvant immunotherapy had significantly better overall survival and disease-free survival compared to those who had surgery within 8 weeks (Fang et al. 2025). Our study showed similar results among those achieved pCR. The reason could be that a prolonged interval may allow for a more mature host immune response and clearance of micrometastatic deposits, which are not immediately apparent on pathology. However, this proposed mechanism remains speculative and requires further validation in prospective studies. Regarding treatment cycles, the optimal number of neoadjuvant immunochemotherapy cycles remains undefined. Rusidanmu et al. reported that three cycles provided greater tumor response and improved DFS compared with two cycles (Rusidanmu et al. 2025), whereas Ye et al. found no significant difference in pCR or survival between two and three cycles (Ye et al. 2025). In our pCR-restricted cohort, patients who achieved pCR after two cycles appeared to have better DFS than those who required three or more cycles. However, this finding should be interpreted with caution, as it is likely subject to confounding by indication—patients receiving additional cycles may represent a subgroup with suboptimal early response or, conversely, excellent response prompting consolidation. Similarly, the association between a shorter treatment-to-surgery interval (≤ 6 weeks) and worse DFS may reflect clinical decisions driven by concerns about progression or poor response, rather than a direct effect of the interval itself. These variables are best viewed as potential markers of higher-risk subgroups rather than direct modifiable causal factors.

This study reviewed patients who achieved pCR after radical esophagectomy and extended lymphadenectomy. However, several limitations should be noted due to its retrospective nature. Firstly, different types of immune checkpoint inhibitors were used in this study, and the impact on survival was unclear due to small study number of each immune checkpoint inhibitor. Secondly, 54 of the 154 patients had treatment in local hospitals, and the pretreatment data including radiological evaluation was unavailable. Thirdly, this study was limited by its single institutional nature. Fourthly, PD-L1 expression data were not consistently available, precluding analysis of its predictive role. However, all procedure was completed with radical lymphadenectomy, and the diagnosis of pCR with inadequate lymph node dissection was excluded from this analysis. Additionally, by restricting the analysis to patients who achieved pCR—a post-treatment outcome—our findings may be subject to collider/selection bias; therefore, they should be considered exploratory and hypothesis-generating rather than confirmatory. Further prospective studies are warranted to validate our findings.

In conclusion, ESCC patients who achieve pCR after neoadjuvant immunochemotherapy have favorable survival outcomes. Advanced pre-treatment tumor stage, an increased number of neoadjuvant treatment cycles, and a shortened treatment-to-surgery interval were identified as factors associated with reduced DFS in this patient population. These findings provide preliminary clinical insights for the individualized management of ESCC patients undergoing neoadjuvant immunochemotherapy, though further prospective validation is needed.

## Data Availability

No datasets were generated or analysed during the current study.
